# Analytical solutions for time-dependent kinematic three-dimensional magnetic reconnection

**DOI:** 10.1371/journal.pone.0286138

**Published:** 2023-05-30

**Authors:** Yalan Chen, Yi Wang, Fengsi Wei, Xueshang Feng, Zilu Zhou, Boyi Wang, Pingbing Zuo, Chaowei Jiang, Xiaojun Xu, Xiaojian Song, Yaxin Gu, Ludi Wang, Xiaoheng Xu, Xinkai Bian

**Affiliations:** 1 Shenzhen Key Laboratory of Numerical Prediction for Space Storm, Institute of Space Science and Applied Technology, Harbin Institute of Technology, Shenzhen, 518055, China; 2 Key Laboratory of Solar Activity and Space Weather, National Space Science Center, Chinese Academy of Sciences, Beijing, China; 3 State Key Laboratory of Lunar and Planetary Sciences, Macau University of Science and Technology, Macau, 999078, China; 4 Shandong Institute of Advanced Technology, Jinan, 250100, China; Central State University, UNITED STATES

## Abstract

Magnetic reconnection is a process that can rapidly convert magnetic field energy into plasma thermal energy and kinetic energy, and it is also an important energy conversion mechanism in space physics, astrophysics and plasma physics. Research related to analytical solutions for time-dependent three-dimensional magnetic reconnection is extremely difficult. For decades, several mathematical descriptions have been developed regarding different reconnection mechanisms, in which the equations based on magnetohydrodynamics theory outside the reconnection diffusion region are widely accepted. However, the equation set cannot be analytically solved unless specified constraints are imposed or the equations are reduced. Based on previous analytical methods for kinematic stationary reconnection, here the analytical solutions for time-dependent kinematic three-dimensional magnetic reconnection are discussed. In contrast to the counter-rotating plasma flows that existed in steady-state reconnection, it is found that spiral plasma flows, which have never been reported before, can be generated if the magnetic field changes exponentially with time. These analyses reveal new scenarios for time-dependent kinematic three-dimensional magnetic reconnection, and the deduced analytical solutions could improve our understanding of the dynamics involved in reconnection processes, as well as the interactions between the magnetic field and plasma flows during magnetic reconnection.

## Introduction

Magnetic reconnection has been proposed to explain the dissipation of magnetic energy in solar flares more than half a century ago [1]. With the development of magnetohydrodynamics (MHD) theory, many authors believe that magnetic reconnection could be described as a topological or geometrical [2] rearrangement process of magnetic field caused by local non-ideal effects induced by the electric field parallel to the magnetic field [3–6]. In the past few decades, the study of magnetic reconnection by theoretical analyses [7,8], numerical simulations [9–11], observations [12–14] and experiments [15–17] has advanced greatly, and we have become more aware of the basic characteristics of magnetic reconnection. Nevertheless, there are still many controversies regarding the processes of energy conversion and the dynamic process in the dissipation region [18–20]. Progress is being made by a combination of analytical models and numerical experiments, but there is still great room for improvement in analytical modelling, especially for the time-dependent three-dimensional (3D) magnetic reconnection.

One of the most important questions in the realm of magnetic reconnection is how the reconnection occurs. Although many theoretical frameworks of magnetic reconnection, such as the Sweet-Parker model [21,22], Petschek model [23], Hall model [24,25] and LV99 model [26] have been proposed that could partially deal with the two-dimensional (2D) reconnection successfully, these models have difficulties in processing the more complicated 3D reconnection [27,28]. Unlike the 2D reconnection in which reconnection can occur only at X-type null points, 3D reconnection allows the process to occur at locations where the field does not vanish and the field lines flip continuously through the plasma rather than being broken at one point [20,29,30].

To mathematically describe the process of magnetic reconnection and the dynamics of the plasma, many scholars have investigated the steady-state solutions in the MHD framework. Due to the nonlinear nature of the coupling between the magnetic field and the plasma, it is very difficult to find analytical solutions. Even if we don’t simplify some equations, such as the momentum equation or the energy equation, direct analytic solutions are still hard to discover. Hence previous analytical methods predominantly simplify the theoretical equations and impose specified constraints. A practicable method is to combine only the Ohm’s law and the simplified Maxwell equations with a particular magnetic field configuration to deduce the analytical solutions. Lau and Finn (30) ignored the magnetic diffusivity and analyzed the reconnection in the singular structures of nulls and closed field lines. In addition, lots of researchers have discussed the magnetic slippage reconnection process in a localized non-ideal region. By assuming specified magnetic field configurations and different forms of local magnetic diffusion, the kinematic solution of the null or non-null magnetic reconnection [31–33], and the flux tube reconnection [34] have been analytically derived. Furthermore, Wilmot-Smith, Hornig [35] built up a completely dynamic reconnection model and deduced the solutions by splitting the variables into a particular non-ideal part and an ideal part. Moreover, by assuming the reconnection is driven by a stagnation-point flow pattern and imposing a series of constraints as well as applying appropriate boundary or symmetry conditions, several authors obtained a family of exact solutions for the steady magnetic annihilation or reconnective annihilation driven by incompressible stationary flow [36–41].

Among the analytical solutions mentioned above, it should be noted that most of the analytical methods have aimed to construct steady-state reconnection models, and only a few studies concerned the cases with a time-dependent magnetic field [42,43]. While the introduced time variable greatly increases the difficulty in analyticity of the equation set, so that specified constraints are imposed to gain probable analytical solutions at the expense of losing universality. Since the dynamic processes related to magnetic field and plasmas will both vary with time in real situations, it is of great interest to seek analytical solutions when reconnection changes over time. In the present paper, based on previous analytical methods for kinematic stationary reconnection, new analytical solutions for time-dependent kinematic three-dimensional magnetic reconnection are obtained and systematically discussed in which essentially the induction equation is considered but the equation of motion is neglected.

### The time-dependent model

The methods used in this paper are built on previous analyses [31], the Maxwell-Faraday equation in our analyses will not be simplified. We start with the Maxwell-Faraday equation and construct a time-dependent model by directly introducing the time variables in the following equations:

E+u×B=ηJ, (1)

$$\nabla \times E=-\frac{\partial B}{\partial t},$$ (2)

∇⋅B=0, (3)

$$\nabla \times B=\mu_{0}J.$$ (4)

Here, Eq (1) is the Ohm’s law, and ***u*** is the velocity of the plasma, *F068* is the magnetic diffusion coefficient, ***J*** is current density, *μ*_0_ is the permeability of the vacuum, ***B*** and ***E*** are the magnetic field and electric field, respectively. The magnetic diffusion coefficient *F068* and magnetic field ***B*** are assumed to vary with time. The introduced time variables increase the difficulty in finding analytical solutions which requires more constraints to be imposed. To keep the universality of the reconnection system as much as possible, we firstly analyze the time-dependent magnetic diffusivity case by letting ∂***B***/∂*t* = 0, and then investigate the time-dependent magnetic field case by assuming ∂*η*/∂*t* = 0.

### The time-dependent magnetic diffusivity case

We adopt the analytical methods for kinematic stationary reconnection which assume an X-type magnetic field in the *x-y* plane superimposed on a uniform field in the *z*-direction [31]. Then we have

$$B=B_{0}(y/Le_{x}+k^{2}x/Le_{y}+e_{z}),$$ (5)

$$J=(k^{2}-1)B_{0}/(L\mu_{0})e_{z}.$$ (6)

Here *k* is a coefficient that determines the magnitude of the electric current, *B*_0_ is the magnitude of the magnetic field, and *L* determines the scale of the system.

Obviously, the electric field can also be expressed as the negative gradient of the electric potential *F066*. Hence, Ohm’s law becomes

−∇ϕ+u×B=ηJ. (7)

The analytic methods used by Hornig and Priest (31) are advantageous to integrating the field lines analytically, which is crucially important in the subsequent analytical processes. Therefore, by following the similar analytic method, we take the scalar product of both sides of Eq (7) with ***B***, and then derive the equation of the field line ***X*** (***x***_0_, ***s***) that passes through an initial point (*x*_0_, *y*_0_, *z*_0_) with its inverse mapping ***X***_0_ (***x***, ***s***). If an appropriate local magnetic diffusion coefficient is given, we can obtain an analytical solution for the electric potential *F066*. Here a time-varying magnetic diffusion coefficient is set to:

$$\eta (x_{0},y_{0},s,t)=\eta_{0}\;exp(-(B_{0}^{2}s^{2}+x_{0}^{2}+y_{0}^{2})/l^{2}-t),$$ (8)

where the parameter *s* is related to the distance of *λ* along the field line by *ds* = *dλ*/|*B*|, and *l* is a constant that governs the scale of a non-ideal region.

In such a diffusive medium, the expression of the electric field can be identified, and the analytical form of the velocity perpendicular to the direction of the magnetic field can be obtained through Ohm’s law. Finally, according to the symmetry condition, the analytical form of the velocity on the *x-y* plane can be obtained:

$$u_{x}=\eta_{0}\sqrt{\pi }(k^{2}-1)\xi_{3}/(2k^{2}lL\mu_{0})exp(-t-\xi_{1})erf(z/l),$$ (9)

$$u_{y}=\eta_{0}\sqrt{\pi }(k^{2}-1)\xi_{2}/(2klL\mu_{0})exp(-t-\xi_{1})erf(z/l),$$ (10)

where *ξ*_1_, *ξ*_2_, and *ξ*_3_ are introduced spatial functions for the conciseness of the expressions:

$$\xi_{1}=({\left(y\;cosh(kz/L)-kx\;sinh(kz/L)\right)}^{2}+{\left(x\;cosh(kz/L)-y/k\;sinh(kz/L)\right)}^{2})/l^{2},$$ (11)

$$\xi_{2}=kx-k^{3}x+k(k^{2}+1)x\;cosh(2kz/L)-(k^{2}+1)y\;sinh(2kz/L),$$ (12)

$$\xi_{3}=y-k^{2}y-(k^{2}+1)y\;cosh(2kz/L)+k(k^{2}+1)x\;sinh(2kz/L).$$ (13)

**Fig 1:**
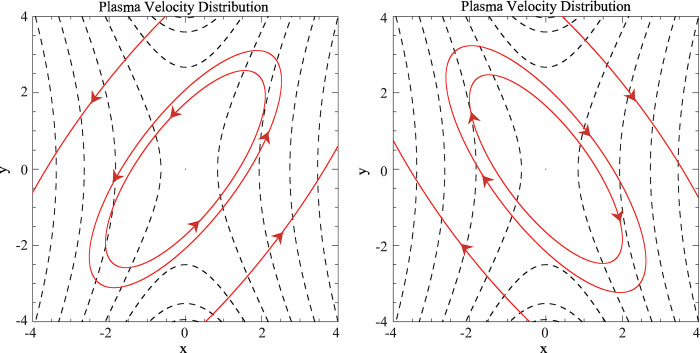
The deduced plasma flows (red solid lines with arrows) and the projection of magnetic field lines (dashed lines): (a) above (*z* = 2) and (b) below (*z* = -2) the reconnection region in the *x*-*y* plane for parameters: *k* = 2, *L* = 10, *l* = 1, *t* = 0.1.

Static models of three-dimensional magnetic reconnection have revealed that as well as the flipping of magnetic flux in the reconnection process [44], the 3D reconnections process will result in the existence of the counter-rotating flow [45]. A counter-rotating flow is a general property in 3D reconnection because of its deep connection with changes of magnetic helicity [46]. Here we use the same parameter as those used in previously stationary work and plot the flow pattern [31]. As shown in Fig 1, the flow velocity also reveals stable counter-rotating flows, which are quite similar to those in the stationary reconnection. Further analyses indicate that the introduced time variable only changes the magnitude of the plasma velocity, but does not change its configuration, hence there is no essential difference in the structure of the flow patterns between the time-dependent magnetic diffusivity case and the previously reported stationary reconnection.

### Time-dependent magnetic field case

To simplify Eqs (1)–(4), the curl of the electric field is zero in Eq (2) for a stationary magnetic field, however, we don’t adopt such simplification in our time-dependent magnetic reconnection cases. Instead, the magnetic field configuration and current density are assumed to have the form:

$$B=B_{0}(y/Le_{x}+k^{2}x/Le_{y}+e_{z})exp(-t),$$ (14)

$$J=(k^{2}-1)B_{0}/(L\mu_{0})exp(-t)e_{z}.$$ (15)

The introduction of time-dependent magnetic field makes Eq (7) no longer hold. The electric field can no longer be represented by the negative gradient of the potential, and correspondingly, we write it as:

$$E=-\nabla \phi -\frac{\partial A}{\partial t}$$ (16)

where ***A*** is the magnetic vector potential, and ***B*** = ∇×***A***. To make Eqs (1)–(4) analytically solvable and to maintain the magnetic field configuration in Eq (14), we construct the following magnetic vector potential:

$$A=(k^{2}xzB_{0}/Le_{x}+B_{0}xe_{y}+B_{0}y^{2}/(2L)e_{z})exp(-t).$$ (17)

Although the basic idea of the analytical method is analogous to those in the time-dependent magnetic diffusivity case, the introduced time-dependent magnetic field makes it much more complicated in the field line integrations ∂X(s,t)/∂s=B(X(s,t)). Here we mainly discuss the variable separated case X(s,t)=X(s)X(t).

In the case of separated variable, according to Eq (14), we can obtain the space segment of the line equations ***X***(*s*,*t*) that passes through an initial point (*x*_0_, *y*_0_, *z*_0_). The components are given as:

$$X=x_{0}cosh(B_{0}ks/L)+y_{0}sinh(B_{0}ks/L)/k,$$ (18)

$$Y=y_{0}cosh(B_{0}ks/L)+kx_{0}sinh(B_{0}ks/L),$$ (19)

$$Z=B_{0}s+z_{0}.$$ (20)

And the corresponding inverse mapping of ***X***_0_ (***x***, ***s***) can be written as:

$$X_{0}=x\;cosh(B_{0}ks/L)-y\;sinh(B_{0}ks/L)/k,$$ (21)

$$Y_{0}=y\;cosh(B_{0}ks/L)-kx\;sinh(B_{0}ks/L),$$ (22)

$$Z_{0}=-B_{0}s+z.$$ (23)

Referring to the previous work, we set *Z*_0_ = 0 and replace *s* = *z*/*B*_0_ in Eqs (21) and (22), and the Euler potentials for the magnetic field with expressions *x*_0_(*x*,*y*,*z*) and *y*_0_(*x*,*y*,*z*) can be obtained respectively. Then, the magnetic diffusion coefficient *F068* also is taken as the form given by Hornig and Priest (31):

$$\eta (x_{0},y_{0},s)=\eta_{0}\;exp(-(B_{0}^{2}s^{2}+x_{0}^{2}+y_{0}^{2})/l^{2}).$$ (24)

Taking the scalar product of both sides of Eq (1) with ***E*** given by (16) with ***B*** and integrating along the field lines, the electric potential can be derived as:

$$\phi =-\int_{0}^{s}(\eta J\cdot B+\partial A/\partial t\cdot B)ds+\phi_{0},$$ (25)

Focus on the mechanism that we are interested in, we set *ϕ*_0_ = 0.

Then, the components of the electric field can be deduced from Eqs (16) and (17):

$$\begin{array}{l} E_{x}=\exp(-2t-\xi_{1})(-B_{0}k^{3}lz\;\exp(\xi_{1})(-(\exp(t)-2)Lx+yz)\mu_{0}\\ \;-(k^{2}-1)B_{0}L\eta_{0}\sqrt{\pi }\xi_{2}/2\;erf(z/l))/(klL^{2}\mu_{0}), \end{array}$$ (26)

$$\begin{array}{l} E_{y}=-\exp(-2t-\xi_{1})B_{0}(2k^{2}l)exp(\xi_{1})(-\exp(t)L^{2}x+Lyz+k^{2}xz^{2})\mu_{0}\\ \;+(k^{2}-1)L\eta_{0}\sqrt{\pi }(-\xi_{3})\;erf(z/l))/(2k^{2}lL^{2}\mu_{0}), \end{array}$$ (27)

$$\begin{array}{l} E_{z}=exp(-2t-\xi_{1}-z^{2}/l^{2})(B_{0}kl(2L\eta_{0}(k^{2}-1)+))L\mu_{0}((\exp(t)-1)y^{2}-2k^{2}x^{2})\\ \;exp(\xi_{1}+z^{2}/l^{2})-4k^{2}\mu_{0}xyz\;\exp(\xi_{1}+z^{2}/l^{2}))-B_{0}(k^{4}-1)\sqrt{\pi }\eta_{0}\;exp(z^{2}/l^{2})\\ \;erf(z/l)\left(-2kxy\;\cosh(2kz/L)+(k^{2}x^{2}+y^{2})sinh(2kz/L)\right))/(2klL^{2}\mu_{0}). \end{array}$$ (28)

To set the *z*-component of the flow to zero, we utilize a flow component parallel to the magnetic field, $v=v_{⊥}-{\left(v_{⊥}\right)}_{z}B/(B_{0}\exp(-t))$, as described by Hornig and Priest (31).

The velocity that is perpendicular to the direction of the magnetic field is derived by:

$$u_{⊥}=\frac{(E-\eta J)\times B}{B^{2}}$$ (29)

To make the derivation process more concise, the following functions are introduced:

$$\varsigma_{1}=-\exp(t)Lx(Ly-k^{4}xz)+z(Ly^{2}+k^{2}xyz-k^{4}x(2Lx+yz)),$$ (30)

$$\begin{array}{l} \varsigma_{2}=(k^{2}-1)\eta_{0}\sqrt{\pi }(-(k^{2}-1)L^{2}y-(k^{2}+1)(L^{2}+2k^{4}x^{2})y\;\cosh(2kz/L))\\ \;+k(k^{2}+1)(L^{2}+k^{4}x^{2}+k^{2}y^{2})x\;\sinh(2kz/L)), \end{array}$$ (31)

$$\begin{array}{l} \varsigma_{3}=(k^{2}-1)\eta_{0}\sqrt{\pi }(kx(k^{2}-1)L^{2}-k(k^{2}+1)(L^{2}+2y^{2})x\;\cosh(2kz/L))\\ \;+\left(k^{2}+1)(L^{2}+k^{2}x^{2}+y^{2})y\;\sinh(2kz/L\right)), \end{array}$$ (32)

$$\begin{array}{c} \varsigma_{4}=(k^{2}-1)\eta_{0}\sqrt{\pi }(\left(k^{2}+1)(k^{4}x^{2}-y^{2})cosh(2kz/L\right)\\ \;-(k^{2}-1)(k^{4}x^{2}+y^{2}+k(k^{2}+1)xy\;\sinh(2kz/L))). \end{array}$$ (33)

And finally, the *x* and *y* components of the velocity can be obtained:

$$\begin{array}{l} u_{x}=\exp(-t-\xi_{1})(-2k^{2}ly\varsigma_{1}\;\exp(\xi_{1})+2k^{2}l(L^{4}x\;\exp(t)-L^{3}yz)\;\exp(\xi_{1})\\ \;+2k^{6}lLx(L\eta_{0}/\mu_{0}\;\exp(-z^{2}/l^{2})(\exp(t)-1)+(Lx^{2}+2xyz)\exp\;(\xi_{1}))\\ \;-k^{4}lL^{2}x(2\eta_{0}/\mu_{0}\exp(-z^{2}/l^{2})(\exp(t)-1)+((\exp(t)-1)y^{2}+2z^{2})\\ \;\exp(\xi_{1}))+(\varsigma_{2}+y\varsigma_{4})L\;erf(z/l))/(2k^{2}lL^{2}(L^{2}+k^{4}x^{2}+y^{2})), \end{array}$$ (34)

$$\begin{array}{c} u_{y}=\exp(-t-\xi_{1})(-2k^{2}lx\varsigma_{1}\;\exp(\xi_{1}))+lL(-2(k^{2}-1)L\eta_{0}/\mu_{0}\;\exp(-z^{2}/l^{2})\\ \;(\exp(t)-1)y-(2k^{2}L^{2}xz(\exp(t)-2))+4k^{2}xy^{2}z-Ly((\exp(t)-1)y^{2})-2\\ \;k^{2}(x^{2}-z^{2}))\;\exp(\xi_{1}))-(\varsigma_{3}/k+x\varsigma_{4})L\;erf(z/l))/(2lL^{2}(L^{2}+k^{4}x^{2}+y^{2})). \end{array}$$ (35)

**Fig 2:**
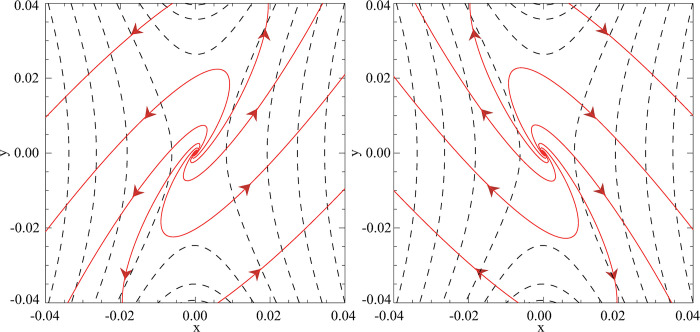
The spiral plasma flows (the solid red lines with arrows): (a) above (*z* = 2) and (b) below (*z* = -2) the reconnection region together with the projection of magnetic field lines in the *x*-*y* plane (the black dashed lines) for parameters: *k* = 2, *L* = 10, *l* = 1, *t* = 0.1.

Fig 2 illustrates that the plasma flows in the ***A*** magnetic vector potential configuration present a distinct spiral distribution which is different from the general reverse rotating flows [47,48].

## Discussion and conclusion

The choice of magnetic vector potentials in Eq (17) is not unique, however, it should be noted that different choices of ***A*** will not affect the overall flow patterns. Assuming two magnetic vector potentials ***A***_**1**_ and ***A***_**2**_, and the electric fields for each vector potential are:

$$\left\{ \begin{array}{l} E_{1}=-\nabla \phi_{1}-\frac{\partial A_{1}}{\partial t}\\ E_{2}=-\nabla \phi_{2}-\frac{\partial A_{2}}{\partial t} \end{array}\right.$$ (36)

Taking the dot product of Ohms law (Eq 25), we can get:

$$\left\{ \begin{array}{l} E_{1}=\nabla \int (\eta J\cdot B+\frac{\partial A_{1}}{\partial t}\cdot B)ds-\frac{\partial A_{1}}{\partial t}\\ E_{2}=\nabla \int (\eta J\cdot B+\frac{\partial A_{2}}{\partial t}\cdot B)ds-\frac{\partial A_{2}}{\partial t} \end{array}\right.$$ (37)

Finally, subtracting one from the other we find:

$$\begin{array}{l} E_{2}-E_{1}=\nabla \int \left(\frac{\partial A_{2}}{\partial t}-\frac{\partial A_{1}}{\partial t}\right)\cdot Bds+\frac{\partial A_{1}}{\partial t}-\frac{\partial A_{2}}{\partial t}\\ \;=\nabla \int \left(\frac{\partial A_{2}}{\partial t}-\frac{\partial A_{1}}{\partial t}\right)\cdot d\lambda +\frac{\partial A_{1}}{\partial t}-\frac{\partial A_{2}}{\partial t}\\ \;=\frac{\partial A_{2}}{\partial t}-\frac{\partial A_{1}}{\partial t}+\frac{\partial A_{1}}{\partial t}-\frac{\partial A_{2}}{\partial t}\\ \;=0 \end{array}$$ (38)

Therefore, different choices for ***A*** will lead to the same electric field, and correspondingly, the same flow patterns.

To reveal the underlying mechanisms responsible for the spiral flows observed in Fig 2, we have further analyzed the flow distributions. The non-ideal flows identified by the previous authors [31,46,49] are confined to field lines that thread through the non-ideal region, however, the spiral flows in Fig 2 are not localized. Thus, the spiral flows may be simply supposed to be a combination of two distinct flows: an expanding flow that is associated with the global reduction in field strength initially assumed by the time-dependent form of ***B***, and a local component that adds a divergence free rotation and is associated with the potential drop along field lines threading the non-ideal region. Based on such speculations, we try to isolate the expanding flow component by setting *η*_0_ = 0 for all equations from Eq (24) onwards, and correspondingly, the new ***u*** components can be deduced as:

$$\begin{array}{l} u_{x}=\exp(-t)(-2Ly(L^{2}+y^{2})z+\exp(t)Lx(2L^{3}-(k^{2}-2)Ly^{2}-2k^{4}xyz)\\ \;+2k^{4}x(L^{2}x^{2}+4Lxyz+y^{2}z^{2})+k^{2}x(-2y^{2}z^{2}+L^{2}(y^{2}-2z^{2})))\\ \;/(2L^{2}(L^{2}+k^{4}x^{2}+y^{2})) \end{array}$$ (39)

$$\begin{array}{l} u_{y}=\exp(-t)(\left(\exp(t)-1)L^{2}y^{3}-2k^{4}x^{2}yz^{2}-2k^{6}x^{2}z((\exp(t)-2)Lx-yz\right)\\ \;-2k^{2}L(\left(\exp(t)-2)L^{2}xz+3xy^{2}z-Ly((\exp(t)-1)x^{2}+z^{2}\right)))\\ \;/(2L^{2}(L^{2}+k^{4}x^{2}+y^{2})) \end{array}$$ (40)

**Fig 3:**
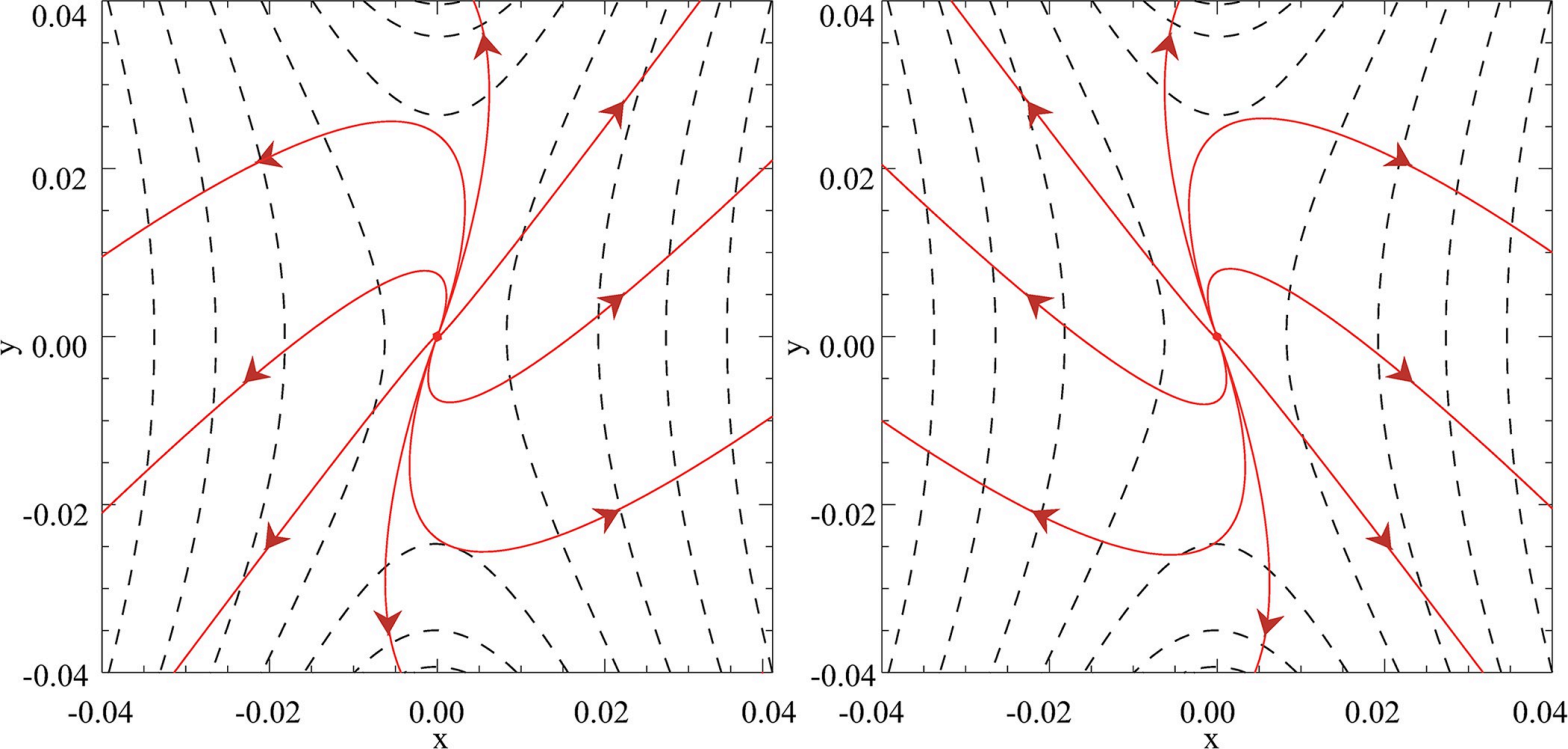
The expanding plasma flows (the solid red lines with arrows): (a) above (*z* = 2) and (b) below (*z* = -2) the reconnection region together with the projection of magnetic field lines in the *x*-*y* plane (the black dashed lines) for parameters: *k* = 2, *L* = 10, *l* = 1, *t* = 0.1.

Fig 3 displays the expanding plasma flows based on Eqs (39) and (40). However, it is noticeable that the expanding flows exhibit a slight rotation in the central region. In addition, if we impose the condition ∂***A***/ ∂*t* = 0 for all equations from Eq (16) onwards and follow the same analytical deductive processes in the time-dependent magnetic field scenario, the deduced flow patterns will be identical to the traditional counter-rotating plasma flows (as shown in Fig 1). Therefore, the newly introduced magnetic vector potential ***A***, along with the remaining terms in ***E*** that arise purely from the time-dependent form assumed for ***B***, should account for the distinct spiral flows in the time-dependent magnetic field case.

Due to the complexity and diversity of the multi-coupling interactions between magnetic field and plasma, analytical solution for three-dimensional magnetic reconnection is one of the most difficult problems in this realm. It has also to be mentioned that, without adding additional constraints other than the referred above, the equation set (1)—(4) cannot be analytically solved if *F068* and ***B*** both vary with time.

In summary, we deduced analytical solutions for time-dependent kinematic three-dimensional magnetic reconnection. Unlike the traditional counter-rotating plasma flows [47,48], it is found that plasma flows in time-varying magnetic reconnection can present new features. Under specified conditions, spiral plasma flows can be generated if the magnetic field changes exponentially with time. Moreover, it will be more complicated to obtain the analytical forms of the field line equations in the variable coupled case, so we simply discuss the variable separated case. While in the simple time-varying magnetic diffusion coefficient case, the plasma flows reveal the previous counter-rotating distributions since the electric potential, the electric field, and the velocity are all linearly coupled with the magnetic diffusion coefficient. Although the above analyses still cannot directly describe the process of energy conversion in magnetic reconnection, these studies could also help improve our understanding of the involved dynamic processes.
